# Robustness and efficiency in interconnected networks with changes in network assortativity

**DOI:** 10.1007/s41109-017-0025-4

**Published:** 2017-03-11

**Authors:** Masaya Murakami, Shu Ishikura, Daichi Kominami, Tetsuya Shimokawa, Masayuki Murata

**Affiliations:** 1 Department of Information Science and Technology, Osaka University, 1-5 Yamada-oka, Suita, Osaka, 560-0871 Japan; 2 Department of Economics, Osaka University, 1-7, Machikane-yama, Toyonaka, Osaka, 560-0043 Japan; 3 Department of Frontier Biosciences, Osaka University, 1-3, Yamada-oka, Suita, Osaka, 560-0871 Japan

**Keywords:** Interconnected network, Assortativity, Brain network, Modular structure, Graph theory, Internet of things (IoT)

## Abstract

In this study, the effect of assortativity on the robustness and efficiency of interconnected networks was investigated. This involved constructing a network that possessed the desired degree of assortativity. Additionally, an interconnected network was constructed wherein the assortativity between component networks possessed the desired value. With respect to single networks, the results indicated that a decrease in assortativity provided low hop length, high information diffusion efficiency, and distribution of communication load on edges. The study also revealed that excessive assortativity led to poor network performance. In the study, the assortativity between networks was defined and the following results were demonstrated: assortative connections between networks lowered the average hop length and enhanced information diffusion efficiency, whereas disassortative connections between networks distributed the communication loads of internetwork links and enhanced robustness. Furthermore, it is necessary to carefully adjust assortativity based on the node degree distribution of networks. Finally, the application of the results to the design of robust and efficient information networks was discussed.

## Introduction

Information networks are characterized by rapid growth and increased complexity. Many sensor devices collect a variety of environmental information and are placed at different locations and connected to the Internet (Atzori et al. 2010; Whitmore et al. 2014). It is estimated that tens of billions of such devices will be connected to the Internet by 2020 (Evans 2011). Additionally, services operated over the network to improve human life will further diversify and the network will be considerably improved to meet changing requirements. Hence, control and management of huge networks, such as the Internet of Things (IoT), will be difficult and involve increasing communication and computational costs. As information networks constitute important infrastructure at present and in the future, high reliability, efficiency, and scalability are important for the control and management of these networks.

The interconnecting structure of multiple networks is important in ensuring the aforementioned properties in information networks. A set of networks with scales that are not too large to be controlled and managed is considered as a large network, which is termed as an interconnected network. The Internet itself is an interconnected network wherein numerous mutually connected networks are operated by Internet service providers (Bailey 1997). Future information networks, including the future Internet, will involve an enormous number of interconnected networks managed by different administrators (Gubbi et al. 2013). It is not possible to guarantee the reliability and efficiency of the network since it is not feasible for a single administrator to manage the entire network. To limit this potential drawback, this study examines the design of interconnected networks to provide high robustness and efficiency.

Structures similar to those in the interconnected networks are observed in networks with modular structures, such as regulatory gene networks, protein-protein interaction networks, and human brain networks (Han et al. 2004). In a modular structure, a module is defined as a subset of network units wherein connections between subset members are denser when compared with connections in the rest of the network. Recent advancements in neuroimaging techniques have allowed the analysis of the human brain at a considerably finer spatial resolution. Thus, extant research has examined the structural network of the brain as represented by anatomical connections among the regions of interest. Previous studies indicated that brain networks possess high topological efficiency and robustness while minimizing wiring cost. Furthermore, the human brain can adaptively tackle a large variety of tasks. It was considered that these advantages were obtained during the process of human growth and evolution.

It is necessary to focus on human brain networks to examine the manner in which interconnected networks can be built. The human brain is a complicated network composed of neuronal cell bodies residing in cortical gray matter regions joined by myelin-insulated axons. Advances in methods to analyze human brains revealed that brain networks include topological features observed in complex networks including small-world properties, a hierarchical modular structure, and an assortative structure (Bullmore and Sporns 2012; Zamora-López et al. 2011; Hagmann et al. 2008). These topological features are considered to provide advantages to the brain such as robustness against node failure and efficiency at tackling tasks adaptively (Bullmore and Sporns 2009). Extant studies discussed applying a human-brain structure to information networks (Klimm et al. 2013).

It is essential to clarify the effects of the structural properties of the human brain to apply the structural properties of the human brain. It is considered that small-world properties of brain networks facilitate efficient communication. However, extant research does not focus on topological advantages of hierarchical modularity. Current opinions on the same are divided. However, hierarchical modularity appears to be associated with communication efficiency, robustness, maintenance of dynamic activity, and adaptive evolution. An understanding of the manner in which the fore-mentioned topological properties contribute to the function of brain networks would contribute significantly to understanding the human brain and the manner in which these properties can be applied.

This study accounted for the modular structure and investigated assortativity, which is defined as the distinctive nodal degree correlation of brain networks. Assortativity represents the degree of correlation between connected nodes. Nodes of similar degree tend to be connected to each other in networks that exhibit high assortativity (termed as assortative mixing). In contrast, in networks with low assortativity (disassortative mixing), nodes are more preferentially connect to each other if they have a larger gap on degree (Brandes 2008). Generally, an assortative-mixing network is robust against selective node failure, and this accelerates the spread of information generated by high-degree nodes (Newman 2002; D’Agostino et al. 2012). Brain networks exhibit a modular structure in which nodes are densely connected to compose a module and modules are sparsely connected with each other. The modules exhibit assortative mixing in this structure. Previous research did not focus on the effect of degree correlation with respect to the formation of edges between modules.

In this study, the effect of assortativity in interconnected networks in terms of robustness and efficiency was investigated. To examine the interaction effects of assortativity within a network and assortativity between networks in detail, networks with different assortativities were constructed and analyzed with respect to the following metrics: (1) edge betweenness centrality (Brandes 2008), (2) average hop length, (3) robustness against node failure, and (4) information diffusion efficiency (Klemm et al. 2012; Bansal et al. 2007).

First, a single network was examined, and the basic properties of assortative networks were demonstrated. This involved constructing a network with a specified value of assortativity by using a rewiring-based method proposed in a previous study (Xulvi-Brunet and Sokolov 2004). To analyze the assortativity between networks, assortativity was defined as the universal assortativity coefficient as proposed in an extant study (Zhang et al. 2012). This was followed by proposing a method with two networks connected such that the assortativity between the networks corresponded to the desired value.

First, we focus on a single network and show the basic properties of assortative networks. For this, we construct a network that has a specified value of assortativity by the rewiring-based method proposed in (Xulvi-Brunet and Sokolov 2004). For analyzing the assortativity between networks, we define assortativity as the universal assortativity coefficient proposed in (Zhang et al. 2012). Then, we propose a method of connected two networks in such a way that the assortativity between them is the desired value.

## Method

### Overview

**Fig. 1:**
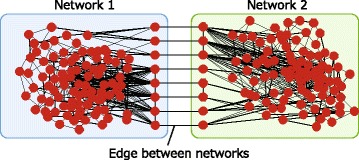
Interconnected modular network

This subsection provides an overview of the method that was used to reveal the effects of assortativity. Two types of assortativity were discussed, namely assortativity within a network and assortativity between networks. In order to examine the influence of these types of assortativity on robustness and efficiency, network construction methods were proposed to achieve the desired assortativity. First, a method to construct a single network with the specific assortativity was discussed. This was followed by proposing a method to construct an interconnected network that consisted of two networks constructed by the first method such that the set of edges connecting the networks yielded the specified assortativity. An example of an interconnected network is shown in Fig. 1.

First, single networks were examined, and the properties of assortativity within a network were demonstrated. This was followed by focusing on interconnected networks to examine the assortativity properties between networks. Additionally, the interaction between within-network assortativity and between-network assortativity was investigated. Networks with different assortativities were constructed to examine the influence of assortativity, and the constructed networks were analyzed relative to graph-theoretic metrics.

Subsection “Definition of assortativity” provides an explanation of the definitions of assortativity within a network and of assortativity between networks. Its subsubsections discusses a method to construct a network with a specific assortativity. Assortativity could influence various metrics and four metrics were used for the evaluation in this study by considering requirements essential to information networks. Subsection “Metrics for evaluation” describes these metrics in detail.

### Definition of assortativity

This subsection focuses on an explanation of the definition of assortativity. Two types of assortativity were defined, namely assortativity within a network and between networks. Assortativity within a network is measured by the *assortativity coefficient* proposed by Newman (Newman 2002). The assortativity coefficient is shown in Subsection “Assortativity within a network”. In contrast, a method to quantify assortativity between networks does not exist to the best of the authors’ knowledge. Hence, the term *universal assortativity coefficient* (Zhang et al. 2012) that corresponds to a dilatation of the assortativity coefficient was used to address this gap. The universal assortativity coefficient is described in Subsection “Assortativity between networks”.

#### Assortativity within a network

Newman proposed measuring the assortativity of a network with the assortativity coefficient (Brandes 2008). The assortativity coefficient is calculated from the remaining degree distribution *q*(*k*) defined as follows:

1 $$ q(k)=\frac{(k+1)p(k+1)}{\sum_{j}jp(j)},  $$

The remaining degree distribution is related to the degree distribution *p*(*k*) that describes the probability that the degree of a randomly chosen node corresponds to *k*. The remaining degree of a node in a path corresponds to the number of edges leaving a vertex separate from the vertex that was arrived along. In other words, the remaining degree of a node in a path is equal to the node’s degree minus one. The joint probability distribution *e*(*j*,*k*) can be introduced given *q*(*k*) wherein the joint probability indicates the probability that two endpoints of a randomly chosen edge have the remaining degrees *k* and *j*. Hence, the assortativity coefficient *r* is defined as follows:

2 $$ r=\frac{1}{\sigma_{q}^{2}}\left[ \sum\limits_{j,\,k}jke(j,k)-\left(\sum\limits_{j}jq(j) \right)^{2} \right],  $$

where *σ*_*q*_ denotes the standard deviation of the remaining degree distribution *q*(*k*), given as follows: $\sigma _{q}^{2} = \sum _{k}k^{2}q(k) - \left (\sum _{j}jq(j)\right)^{2} $. The range of values that *r* can belong to corresponds to [−1, 1]. Positive and negative values of *r* indicate an assortative network and a disassortative network, respectively. When *r* corresponds to zero or is near zero, nodes are randomly connected with each other independent of their degrees. The range of feasible values of *r* is based on the degree distribution.

#### Assortativity between networks

Universal assortativity coefficient proposed in a previous study (Zhang et al. 2012) was used to define the assortativity between networks. This coefficient reflects the contribution of an individual edge’s to the global assortativity coefficient of the entire network. This was used to analyze the assortativity of any part of a network in a previous study (Zhang et al. 2012). The universal assortativity coefficient for a set of targeted edges *E*_*target*_ is represented as the sum of the contribution of each edge to the assortativity of the entire network as described in the previous subsubsection. The contribution of each edge to global assortativity is based on the global assortativity *r* in Eq. 2. Global assortativity *r* can be expressed as follows:

3 $$ r=\frac{1}{\sigma_{q}^{2}}\left(E\left[\left(\,J-U_{q}\right)\left(K-U_{q}\right)\right]\right),  $$

where $U_{q}=\sum _{j}jq(j)$ denotes the expected value of the remaining degree, and *J* and *K* denote variables of the remaining degree, which have the same expected value *U*_*q*_. Then, the contribution *ρ*_*e*_ of the edge *e* is then defined as follows:

4 $$ \rho_{e}=\frac{\left(\,j-U_{q}\right)\left(k-U_{q}\right)}{M\sigma_{q}^{2}},  $$

where *M* denotes the number of edges in the whole network, and *j* and *k* denote the remaining degrees of the two endpoints of the edge *e*. Finally, the universal assortativity coefficient *ρ* is defined as follows:

5 $$ \rho=\sum\limits_{e\in E_{target}} \rho_{e}=\sum\limits_{e\in E_{target}} \frac{\left(\,j-U_{q}\right)\left(k-U_{q}\right)}{M\sigma_{q}^{2}}.  $$

The universal assortativity coefficient *ρ* is a part of global assortativity. Thus, if *E*_*target*_ is considered as the set of all edges, then *ρ* is equal to Newman’s global assortativity. In this study, each edge between networks corresponds to an element of *E*_*target*_. The assortativity between networks is calculated from Eq. (5) based on the *E*_*target*_.

### Network construction methods for different assortativities

This subsection presents two methods of constructing a network. The first subsubsection presented a method of constructing a single network that included the specified assortativity within the network as proposed in a previous study. The second subsubsection includes the proposal of a method to construct an interconnected network with the specified assortativity between two component networks.

#### Single networks with different assortativities within a network

A network with a target assortativity was constructed by repeatedly rewiring the edges of a given network. In this method, the assortativity was changed without changing the degree distribution because the individual rewirings did not change the degree distribution. Although this rewiring method did not necessarily achieve the overall maximum or minimum assortativity, this method has been widely used in previous researches relevant to assortativity due to its low computational cost (Xulvi-Brunet and Sokolov 2004; Van Mieghem et al. 2010; Noldus and Van Mieghem 2013), and it was also discussed that rewiring method can well approximate optimal solutions (Winterbach et al. 2012).

**Fig. 2:**
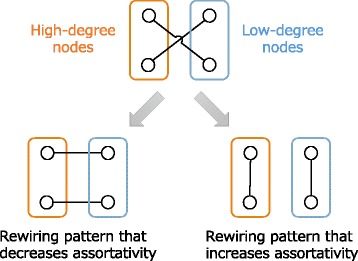
Rewiring patterns: decreasing assortativity (*left*) and increasing assortativity (*right*)

Repeated rewiring of the edges in the network proceeded in the following manner. First, two edges that did not share a common endpoint were randomly selected. This was followed by selecting four nodes as the new endpoints for the edges. Two pairs of nodes were rewired such that *r* approached the desired value, as shown in Fig. 2. In the rewiring process, only the degree was considered to determine the nodes that should be connected with each other. Two nodes with degrees that exceeded the degrees of the other nodes were wired to increase assortativity. This contrasted with both the previously mentioned rewiring methods that decreased assortativity. Increasingly effective patterns to decrease assortativity were selected by calculating both assortativity coefficients. It should be noted that the two initially selected edges were not rewired when the connection pattern without rewiring was the most suitable and when the rewiring disconnect the network.

#### Inter-connected network with different assortativities between networks

Interconnected Network with Different Assortativities Between Networks Two identical networks were connected to each other by *M* edges to construct an interconnected network with the specified assortativity between networks. In order to obtain a suitable mixing pattern, edges between the networks were repeatedly deleted and added. This was performed stochastically with the mixing pattern determined by the following procedure:

1. Two networks were randomly connected via *M* edges. At this time, an endpoint node did not have multiple edges.
2. The assortativity between the networks was calculated. If the assortativity between the networks corresponded to the target value, then the set of connections at this point was adopted. Otherwise, the following steps were repeated until the assortativity between the networks reached the desired value.
3. An edge between the networks was randomly selected and deleted.
4. An edge was randomly added between the networks. If the assortativity did not approach the target value to a closer extant than the prior assortativity value, then the added edge was deleted, and the edge deleted in step 3) was re-added. The selection of an additional link was then repeated.
  In contrast with assortativity within a network, edges that influence assortativity could arbitrarily be chosen free from any degree constraints. Therefore, the maximum or minimum values of assortativity between networks for given networks were easily calculated.

### Metrics for evaluation

The metrics for the evaluation included the following: (1) the edge betweenness centrality, (2) the average hop length, (3) robustness relative to node failure, and (4) information diffusion efficiency. The details of these metrics are described in the following subsections.

**Edge betweenness centrality** The edge betweenness centrality of a network is defined as the number of shortest paths that passed through an edge in the network (Brandes 2008). This could be considered as the communication load on the edges, and it indicated a possible concentration of the communication load. In the context of information networks, edges with high edge betweenness centrality were associated with a higher probability of experiencing traffic congestion.

**Average hop length** The average path length corresponds to the average of the hop count of all shortest-hop paths. This is used widely in the field of graph theory and can be used to characterize data-transfer efficiency. Since we define the information diffusion efficiency in a network as a speed that information is diffused throughout the network in a probabilistic model for information diffusion, a network that has a small average hop length achieves a high information diffusion efficiency.

**Robustness** With respect to single networks, robustness was evaluated by using giant component size following the removal of a few nodes. The giant component size is the number of nodes in the largest connected component. Networks that maintained a high giant component size were considered to possess higher connectivity and consequently robustness.

With respect to the robustness of interconnected networks, it is inappropriate to use the giant component size because only a limited number of nodes include edges between networks, and the removal of other nodes corresponds to a considerably small influence on the performance of assortativity between networks. Removing nodes with the fore-mentioned interconnected edges could constitute a future research topic. However, all the nodes remain connected until all interconnecting edges are removed unless the endpoint nodes include extreme bottlenecks within the corresponding belonging network. Therefore, in order to evaluate the effect of interconnecting edges, average hop length between networks was used while removing endpoint nodes of the edges. With respect to the average hop length, paths between two nodes in the same network were ignored because they were barely influenced by the edges between networks. Thus, an interconnected network was considered robust if it retained its original value of average hop length following the removal of a few endpoint nodes.

**Information diffusion efficiency** The susceptible-infected-recovered (SIR) model in a previous study (Kermack and McKendrick 1927) was used to model the diffusion of information. In this model, each node could belong to three states, namely susceptible (S), infected (I), and recovered (R) state. An infected node transmitted infection to neighbor nodes with probability *β*, and recovered with probability *γ*. A recovered node did not infect nor pass its infection to other nodes. When *γ*=0 in the SIR model, it was termed as a susceptible-infected (SI) model (Antman et al. 1993) wherein when a node in a network was infected, all other susceptible nodes converged into an infected state. This SI model could purely measure the information diffusion speed of a network, i.e., the degree to which a network can diffuse information. Conversely, when *γ*>0, all infected nodes eventually recovered and some nodes remained susceptible. In this case, areas that were more likely to be infected could be detected after a number of simulations selected an initial infected node randomly.

Several other models could be used to simulate the diffusion of information. However, the SIR model was used in the present study for two reasons. First, it was widely used in modeling the diffusion of information. Second, it offered immediate convergence.

## Results

### Single networks

In this study, single networks with different assortativities were investigated. This involved using two types of networks that have different nodal degree distribution. The first type corresponded to a scale-free network (SF network) whose degree distribution follows power-law. Power-law distribution has been found in many complex networks, such as airline networks, social networks, the Internet, and so on. This type of networks is generated by two steps. First, we generate Barabási-Albert networks (Barabási and Albert 1999) which leads to the degree distribution *p*(*k*)∼*k* ^−3^. Second, we keep its degree distribution and rewire all edges so that the characteristics of Barabási-Albert networks do not affect our evaluation. A single SF network consists of 100 nodes and 295 edges in which each node degree corresponded to a minimum of 3. Networks with different assortativities were generated by rewiring the edges of this network as described in the previous section. Assortativities within a range of −0.69≤*r*≤0.58 could be obtained by rewiring the edges of this network.

The second type corresponded to a Erdös-Eényi random network (RN network). The degree distribution of the RN network followed a Poisson distribution that is similar to the distribution observed in wireless sensor networks. A single RN network consists of 100 nodes and 300 edges. The network was generated by repeatedly selecting pairs of nodes at random and connected these pairs. For this type of network, assortativities in the range of −0.79 and −0.97 were obtained. It was observed that the assortativity range of an SF network was narrower than that of a RN network, this reflected that the distribution of node degree was more strongly biased in SF networks. In particular, the number of nodes of high degree was lower, and thus, such nodes were rarely connected to other nodes of the same degree, thereby decreasing assortativity.

In the following results for single networks, we construct 25 topologies for each value of *ρ*; the results shown below are the averages across all 25 topologies.

#### Average hop length

**Fig. 3:**
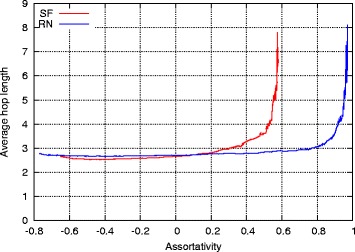
Average hop length in single networks with different assortativities

The relation between average hop length and assortativity is shown in Fig. 3. Both SF and RN networks exhibited the same tendency except with respect to the range of assortativity. As shown in Fig. 3, the average hop length value increased when *r* increased. Specifically, the average hop length value rapidly increased when *r* approached its highest value for each network. With respect to information networks, an increase in the value of average hop length often degraded performance by increasing communication delays.

**Fig. 4:**
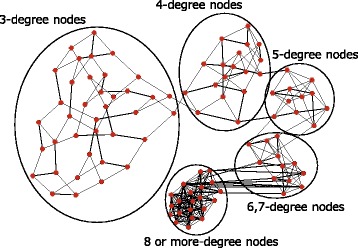
SF network topology with high assortativity (*r*=0.58)

It was important to identify the reason for the sudden increase in average hop length when assortativity approached its highest value. This was performed by considering SF topology with assortativity *r*=0.58 that corresponded to the peak. Figure 4 shows this network. Additionally, these clusters could be organized in order of degree. In this topology, almost all nodes were connected to other nodes of the same degree, and thus sets of same-degree nodes formed clusters. The results indicated that RN networks exhibited the same tendency. A chain-like topology possessed a high average hop length when compared with that of small-world topology, and thus a highly assortative topology implied a higher average hop length value. This tendency grew stronger as the assortativity of the network increased.

Hence, it was concluded that average hop length rapidly increased as assortativity approached its maximum value mainly because there were fewer shortcut edges and a few shortcut edges can markedly reduce average hop length, as demonstrated in a previous study (Newman 2000). However, these types of shortcut edges are likely to be lost when assortativity is very high because a clustered topology of the type shown in Fig. 4 emerged. The observations indicated that the network lost these shortcut edges when the value of *r* was very high. Consequently, average hop length rapidly increased as assortativity approached a maximum value.

#### Edge betweenness centrality

**Fig. 5:**
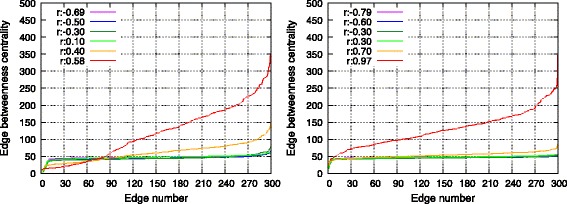
Edge betweenness centrality of all edges in a single SF network (*left*) and in a single RN network (*right*)

Figure 5 shows edge betweenness centrality of each edge in a single SF and RN network. As shown in the figure, edges were arranged along the *x*-axis in increasing order of edge betweenness centrality. The figure indicated that with respect to the topology with maximum *r* (1), a few edges indicated extremely high edge betweenness centrality, and (2) the total edge betweenness centrality was also considerably high. This was attributed to the clustered structure shown in Fig. 4. There were also very few shortcut edges in this topology, and the load on these edges increased. The lack of shortcut edges also caused an increase in the average hop length value as shown in the previous section. Therefore, edges were included in the shortest paths several times. With respect to the other topologies, the total communication load over all edges drastically decreased due to the emergence of shortcut edges. Furthermore, it was observed that the distribution of edge betweenness centrality became more homogeneous as the assortativity decreased. This was because the connections between high-degree nodes changed into connections between a high-degree node and a low-degree node, and the communication load was distributed. Specifically, single RN networks with minimum assortativity *r* completely distributed the edge betweenness centrality since its node degree followed a Poisson distribution in contrast to SF networks that possess a Power law distribution.

Table 1 summarizes the relation between edge betweenness centrality and degrees of the endpoints of the edges in a single SF network. High-degree nodes are important for a communication load in single disassortative networks, while low-degree nodes that connect different clusters are important in single assortative networks.

**Table 1:** Relation between edge betweenness centrality and degrees of endpoints of edges in a SF network. Only 5 out of 295 edges that correspond to the highest or lowest edge betweenness centrality are selected

Most disassortative			Most assortative		
(*r*=−0.69)			(*r*=0.58)		
EBC	Endpoint 1	Endpoint 2	EBC	Endpoint 1	Endpoint 2
8.26	7	7	1.00	13	13
9.70	7	7	1.00	15	15
11.7	7	7	1.00	8	8
15.0	7	7	1.00	13	13
15.1	7	7	1.00	13	13
⋮					
83.0	25	6	1070	6	5
88.8	19	6	1078	6	7
97.7	13	7	1136	4	4
100	15	7	1421	4	3
168	25	7	1455	5	4

#### Robustness

**Fig. 6:**
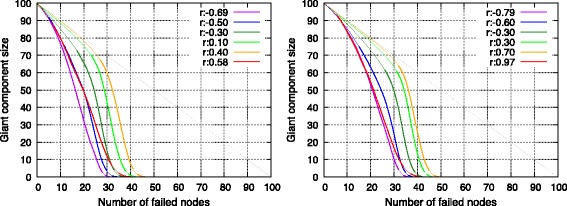
Change in giant component size with respect to deliberate node failure in a single SF network (*left*) and in a single RN network (*right*)

**Fig. 7:**
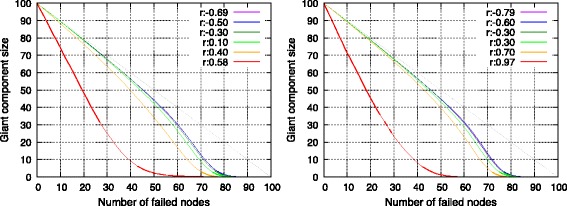
Change in giant component size with respect to random node failure in a single SF network (*left*) and in a single RN network (*right*)

Figures 6 and 7 show the change in giant component size of networks when nodes are removed from the highest degree node either at each step or randomly. Simulations of removal were run 50 times with respect to each topology. With the exception of networks with maximum assortativity, assortative topologies were robust with respect to selective failure and weak with respect to random failure. As explained in the previous section, an assortative topology consisted of clusters connected in a chain. In the selective node-failure scenario, node failure commenced at the high-degree side of this chain. Therefore, nodes with lower degrees remain connected to each other even when high-degree nodes failed. Conversely, the probability for the breaking of edges between clusters in the chain increased if the sequence of node failure did not follow any order. Thus, assortative topologies were fragile with respect to random node failure.

Robustness with respect to selective failure suddenly decreased when assortativity reached a maximum value. This corresponded to a topology with a minimum number of edges between clusters because the edges between clusters connected nodes of differing degrees. Thus, connectivity between clusters was fragile with respect to selective node failure.

An interesting point was that single RN networks exhibited considerably lower robustness when compared with that of SF networks given that both networks possessed maximum values of assortativity. This difference reflects the difference in the mode of degree. Many nodes in an SF network possess a minimal degree and they construct a cluster. Thus, selective node failure does not divide the giant component until additional nodes fail.

The aforementioned observations indicated that single assortative networks were robust with respect to selective failure albeit not with respect to random failure. However, when the fore-mentioned networks were extremely assortative, then generated networks were weak with respect to both types of failures.

#### Information-diffusion efficiency

**Fig. 8:**
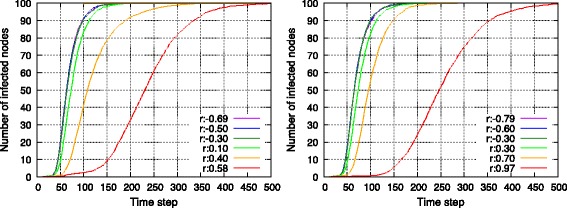
Information diffusion speed in a single SF network (*left*) and in a single RN network (*right*)

As shown in Fig. 8, speed of information diffusion is measured using a SI model with *β* set to 0.05. The *x*-axis represents time steps of diffusion, and the number of infected nodes is counted on the *y*-axis at each time step. Simulations of diffusion were executed 50 times with respect to each topology. A shown in Fig. 8, information diffused poorly when the topology possessed high assortativity in both SF and RN networks. With respect to an assortative network, the low- and high-degree nodes involved few connections between them, and this resulted in a network with low efficiency of information diffusion.

**Fig. 9:**
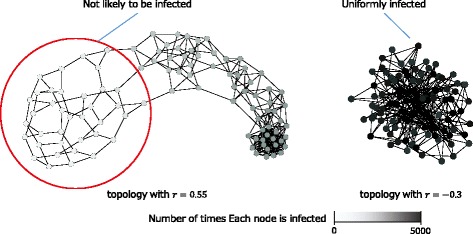
Number of infections of each node

Additionally, SIR models of *β*=0.05 and *γ*=0.10 were used to identify the areas that were more likely to be infected, and the number of infections on every node until a diffusion converged was counted. It should be noted that the SI model did not match this measurement because the infected nodes propagated infection permanently. Figure 9 shows the number of infections of each node in the SF topologies with *r*=0.55 and −0.30. The total number of simulations run for each topology corresponded to 5,000 (50 simulations for every 100 node). All nodes were uniformly infected with respect to the topology with *r*=−0.30. In contrast, with respect to the assortative topology with *r*=0.55, it was not likely that the information would diffuse in sparse areas and stack in high-degree dense areas.

### Interconnected networks

In this subsection, interconnected networks with differences between-network assortativities *ρ* were investigated. Each interconnected network consisted of two single networks of the same type that were connected to each other. Thus, a single SF network based on Barabási-Albert model included 1000 nodes and 2995 edges. Additionally, a RN network based on Erdös-Rényi model included 1000 nodes and 3000 edges. Two single networks of the same type were connected to each other via *M* edges, i.e., SF-SF networks and RN-RN networks.

**Fig. 10:**
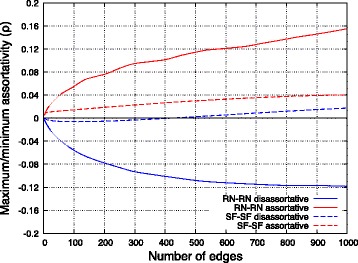
Relationship between the number of edges between networks and the maximum and minimum values of assortativity between networks

Before starting the evaluation, it was important to clarify the appropriate value of *M* to measure the effect of changing assortativity *ρ*. Figure 10 shows the relationship between the number of edges between networks and the maximum and minimum values of assortativity *ρ*. In this figure, edges were added in order of the influence on assortativity *ρ*. First, the range of *ρ* for RN-RN networks exceeded that of SF-SF networks since a SF network included a wider range of node degrees. However, it did not necessarily mean that larger limit of assortativity caused larger effect on the performance of RN-RN networks. We showed details about this point in the following evaluation of interconnected networks. Second, we also found that only a small fraction of edges could considerably increase or decrease assortativity *ρ*. Increment of all of the four curves was the largest at first, and as adding more edges the curves became gentler. To our surprise, the curve of SF-SF disassortative went so far as to cross the *x*-axis. This was because the average degree of a SF network is almost double of that of a RN network. Therefore, SF-SF networks has fewer combination of endpoints that contributes to increase disassortativity. Considering the above, *M* was set to 50 in the following evaluation.

The range of assortativity *ρ* between the networks was from −0.0054 to 0.0124 for SF-SF networks and from −0.037 to 0.0385 for RN networks. In the evaluation, the target assortativity *ρ* was changed, and edges were generated between networks. The construction method for interconnected networks included a probabilistic process. Thus, 25 topologies for each value of *ρ* were constructed and the results shown below correspond to the averages across all 25 topologies.

#### Edge betweenness centrality

**Fig. 11:**
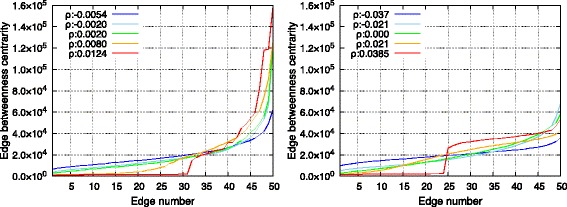
Edge betweenness centrality of every edges in SF-SF networks (*left*) and in RN-RN networks (*right*)

Figure 11 shows the edge betweenness centrality of interconnecting edges. The edge betweenness centrality could be considered as the communication loads on edges. In a manner similar to the single networks shown in Fig. 5, disassortative edges between the networks could distribute communication loads in both SF-SF and RN-RN networks. All edges between networks connected high-degree nodes with low-degree nodes when networks were connected disassortatively. Therefore, communication loads were distributed to all edges between networks.

In contrast, topologies with larger *ρ* exhibited different and interesting properties when compared with those of single networks. First, edge betweenness centrality on the edges were more biased as *ρ* increased in single networks. However, a heavy communication load was assigned to edges with low-degree nodes in single assortative networks, as summarized in Table 1. In contrast, in interconnected networks, edges between the high-degree nodes carried a heavy communication load as shown in Tables 2 and 3 because those nodes were accessible to other nodes in each network. Thus, features on node degrees of edges that possessed high edge betweenness centrality differed between single and interconnected networks. Another interesting property was that there was a sudden increase of edge betweenness centrality in the topologies with extremely high *ρ*. This was because the types of interconnecting edges were clearly separated into connection between high-degree nodes and connection between low-degree nodes. The ratio of increase in the edge betweenness centrality was more significant in SF-SF networks due to the power-law distribution of node degree. The relation between edge betweenness centrality and node degree is confirmed in Tables 2 and 3.

**Table 2:** Relation between edge betweenness centrality and degrees of endpoints of the interconnecting edges in a SF-SF network. Only 5 out of 50 edges with the highest or lowest values of edge betweenness centrality are selected

Most disassortative			Most assortative		
(*ρ*=−0.0054)			(*r*=0.012)		
EBC	Network 1	Network 2	EBC	Network 1	Network 2
5.6×10^3^	36	7	1.2×10^3^	4	4
7.3×10^3^	7	24	1.3×10^3^	4	4
8.2×10^3^	26	7	1.3×10^3^	4	4
9,2×10^3^	22	7	1.4×10^3^	4	4
1.0×10^4^	7	22	1.4×10^3^	4	4
⋮					
3.3×10^4^	49	4	6.1×10^4^	51	51
3.4×10^4^	87	4	6.6×10^4^	59	59
4.8×10^4^	4	104	9.0×10^4^	65	65
6.6×10^4^	86	4	1.3×10^5^	87	87
1.0×10^5^	104	4	1.7×10^5^	104	104

**Table 3:** Relation between edge betweenness centrality and degrees of endpoints of the interconnecting edges in a RN-RN network. Only 5 out of 50 edges with the highest or lowest values of edge betweenness centrality are selected

Most disassortative			Most assortative		
(*ρ*=−0.036)			(*r*=0.037)		
EBC	Network 1	Network 2	EBC	Network 1	Network 2
8.0×10^3^	2	13	1.7×10^3^	2	2
1.1×10^4^	14	2	1.8×10^3^	2	2
1.1×10^4^	14	2	1.8×10^3^	2	3
1.1×10^4^	2	13	2.1×10^3^	2	2
1.2×10^4^	13	2	2.1×10^3^	3	2
⋮					
2.8×10^4^	3	12	3.2×10^4^	13	13
3.0×10^4^	13	2	3.3×10^4^	13	14
3.1×10^4^	2	17	3.3×10^4^	13	14
3.3×10^4^	2	13	3.7×10^4^	17	13
4.1×10^4^	3	12	4.3×10^4^	15	15

#### Robustness

With respect to the evaluation of robustness of interconnected networks, endpoint nodes of interconnecting edges were deliberately or randomly broken, and average hop length between networks was calculated. In this context, average hop length did not include paths between two nodes in the same network. Simulations of removal were executed 25 times with respect to each topology.

**Fig. 12:**
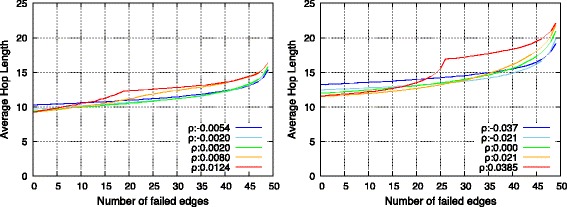
Change of average hop length against deliberate failure on endpoints of inter-connecting edges in SF-SF networks (*left*) and in RN-RN networks (*right*)

**Fig. 13:**
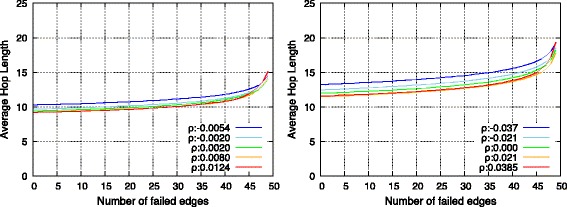
Change of average hop length against random failure on endpoints of inter-connecting edges in SF-SF networks (*left*) and in RN-RN networks (*right*)

Figures 12 and 13 show the results, and it was observed that SF-SF and RN-RN networks exhibited almost identical tendencies. First, prior to checking the robustness, it was observed that the average hop length decreased with topologies with high *ρ*. The main reason for this corresponded to the connections between high-degree nodes. As observed in the previous section, assortatively interconnected networks involve several connections of high-degree nodes that include a high communication load. Therefore, they contribute to a decrease in the average hop length.

With respect to the selective failure shown in Fig. 12, assortative networks possess lower average hop length, and are therefore initially efficient. However, subsequently as endpoint nodes were selectively removed, there was a performance reversal between assortative and disassortative networks. This could be attributed to the loss of connections between high-degree nodes. Conversely, as shown in Fig. 13, the performance of average path length simply decreased while maintaining the order of assortativity.

**Fig. 14:**
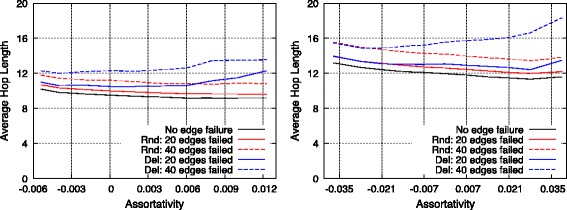
Relation between average hop length and assortativity between networks in SF-SF networks (*left*) and in RN-RN networks (*right*)

In summary, as shown in Fig. 14, topologies with low *ρ* were tolerant of both random and selective failure, while topologies with high *ρ* provided efficient average hop length and were tolerant of random failure albeit vulnerable to selective failure.

#### Information-diffusion efficiency

**Fig. 15:**
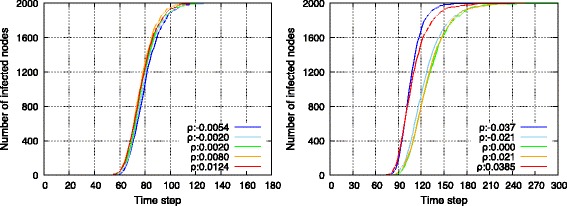
Information diffusion speed in SF–SF networks (*left*) and in RN–RN networks (*right*)

Figure 15 shows the speed of information diffusion. Interestingly, SF-SF networks and RN-RN networks behaved differently. Assortative topologies spread information slightly faster in SF-SF networks. This was probably due to powerful highest-degree nodes termed as *hubs* in SF networks. However, a SF network itself constituted an efficient network with respect to information diffusion, and thus differences between topologies with different *ρ* were small. More interestingly, with respect to RN-RN networks, the efficiency of assortative and disassortative networks exceeded that of non-assortative networks. This could be because both assortative and disassortative interconnected networks involved edges with high-degree nodes, while non-assortative networks did not. Another interesting point was that although low assortativity led to a low performance in terms of average hop length, it also caused fast information diffusion. As summarized in Table 3, endpoints of edges in disassortative RN-RN network homogeneously contained high-degree nodes, and this property could help in the diffusion of information over all the nodes. These results indicated that information-diffusion efficiency could not be uniquely defined by assortativity between networks and instead depended on types of each network.

## Discussion

### Effect of assortativity on robustness and efficiency

With respect to single networks, the results of the present study indicated that an increase in assortativity caused the following

1. High average hop count
2. High robustness relative to the failure of high-degree nodes
3. Low robustness relative to random node failure
4. Low efficiency of information diffusion
5. Concentration of communication loads on the edges connecting clusters

It should be noted that disassortative networks exhibited opposite features, i.e., a decrease in assortativity caused a low hop count, high information diffusion efficiency, and distribution of communication load. An intuitive explanation of the effects as detailed in the aforementioned points 1-5 was that high assortativity resulted in fewer shortcut links between high-degree nodes and low-degree nodes and formed a chain-shape network. It should be noted that extremely high assortativity led to fragile connectivity. The results indicated that the assortativity of a network should be within an appropriate range of values.

With respect to interconnected networks, the results revealed that an increase in assortativity led to the following:

1. Low average hop count
2. Low robustness against the failure of high-degree nodes
3. Normal robustness against random node failure
4. High efficiency of information diffusion
5. Concentration of communication loads on edges connecting hub nodes

In this case, disassortative networks exhibited opposite features with the exception of the efficiency in information diffusion (it also exhibited high efficiency). The results also indicated that the performance of assortativity between networks was based on the degree distribution of each network. For example, communication loads of interconnecting edges were considerably biased when networks possessed power-law of degree distribution. The range of assortativity was also influenced by the degree distribution. Thus, the fore-mentioned results characterized the relations among robustness, efficiency, and assortativity within a network.

### Assortativity in brain networks

In the study, assortativity in interconnected networks was evaluated, and the effect of assortativity on robustness and efficiency was demonstrated. In this section, the results of the present study were compared with those related to the assortativity of human brain networks as obtained in our previous study. Here, we briefly summarize the result of the study. We used datasets of human brain networks from (Hagmann et al. 2008). These datasets contain the weighted connections between the regions of interests (ROI) in a brain. The number of ROIs is 998. We used a threshold on the weight of connections to define an undirected edge between ROIs.

**Fig. 16:**
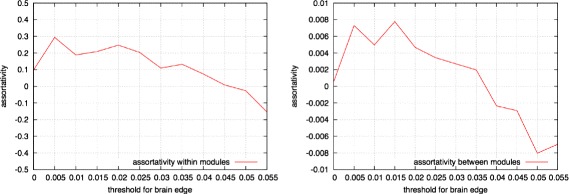
Average assortativity within ROIs (*left*) and between ROIs (*right*) in brain networks

We measured the average assortativity within the ROIs and the average assortativity between ROIs using the Louvain method (Blondel et al. 2008) to identify the modular structure. Figure 16 show the results for assortativity within and between ROIs. With respect to within-module...(omission)...hop length was reduced.

With respect to human brain networks, the network exhibited between-module assortativity when both strong and weak edges were included. This indicated that the human brain could communicate efficiently between modules. In contrast, modules were connected disassortatively when only strong edges were considered. This could facilitate concurrent processing between two modules. In order to design information networks, different values for assortativity were used based on the edge importance.

### Information-network design with the consideration of assortativity

The effect of assortativity on the robustness and efficiency of interconnected networks was examined. The correlation of the assortativity with only the robustness and efficiency of a network were demonstrated previously, and thus assortativity could not be used to determine whether or not a given network was robust and efficient. However, it was important to discuss the construction of a new network such that it possessed good robustness and efficiency. Networks aimed at disseminating information should be constructed such that they possess low assortativity. In contrast, low assortativity was desirable to construct networks that could spread data quickly. Furthermore, when multiple networks were integrated, the results indicated that the assortativity between networks could be adjusted to control the trade-off between efficiency and load balancing.

For an example, an ad-hoc network composed of IoT devices was considered as an application of the results of the present study. In this case, the entire network was composed of ad-hoc networks that were in turn composed of homogenous devices. The construction of an assortative network has various advantages in this case and include high robustness and good robustness with respect to computer-virus infections. This type of an assortative network also included disadvantages such as high average hop length and a concentration of communication loads. However, the average hop length of the network was not exceedingly high if the assortativity of the network was not too high. Thus, an appropriate setting for the assortativity is important.

A detailed method for constructing an assortative ad-hoc network is beyond the scope of this study. However, this could be achieved by selecting node deployment techniques and transmission-power control techniques. For example, nodes with similar degrees are likely to be connected when more nodes are arranged near the center of the field. Constructing shortcut links between nodes with similar degrees also contributed toward an assortative network and reflected cases in which nodes used directional beams or long-range omnidirectional transmission.

The following advantages were observed when networks were connected with each other assortatively. The connections between high-degree nodes reduced the average hop length. Connections between low-degree nodes improved the robustness relative to the failure of high-degree nodes. The type of network failures considered here reflected the depletion of electric power that resulted from the concentration of communication. Communication loads are distributed if networks are connected disassortatively.

## Conclusion and future work

In this study, the effect of assortativity on the robustness and efficiency of interconnected networks was examined. With respect to the assortativity of single networks, it was observed that an increase in assortativity caused (1) an increase in the hop count, (2) high robustness of connectivity with respect to the failure of high-degree nodes, (3) low robustness of connectivity with respect to random node failure, (4) low efficiency for information diffusion, and (5) concentration of communication loads on a few edges. Simultaneously, these results implied that single disassortative networks involved opposite features. It was also observed that an increase in assortativity reduced the shortcut links in networks. Therefore, an excessive increase in assortativity harmed the network in terms of communication efficiency, robustness, and communication load on network links. With respect to the assortativity between networks, the following results were observed: (1) a decrease in the hop count, (2) low robustness of connectivity with respect to the failure of high-degree nodes, (3) normal robustness of connectivity with respect to random node failure, (4) high efficiency with respect to information diffusion, and (5) concentration of communication loads on a few edges.

Additionally, it was observed that the performance of assortativity between networks depended on the degree distribution of each network. Although, the results of this study demonstrated the effect of assortativity on the robustness and efficiency of interconnected networks, the results were only applicable to networks involving the Barabási-Albert model and Erdös-Rényi random network model. The study also investigated assortativity in the case of networks with nodes of uniform degree. However, interconnected networks composed of networks with various degree distributions were not investigated, and this will be the subject of a future study.

The study also discussed methods to construct a network that was robust and efficient. In actual networks, various constraints affect the construction of assortative or disassortative networks. For example, with respect to ad-hoc networks of wireless sensor devices, it is necessary to consider the battery life of sensors and communication distances. This study did not propose a model to generate a network topology, and thus, a future study will include the proposal of a generation model for assortative or disassortative networks and the application in an actual network.
